# Label-free CARS microscopy reveals similar triacylglycerol acyl chain length and saturation in myocellular lipid droplets of athletes and individuals with type 2 diabetes

**DOI:** 10.1007/s00125-020-05266-6

**Published:** 2020-09-03

**Authors:** Sabine Daemen, Anne Gemmink, Alexandra Paul, Nils Billecke, Katrina Rieger, Sapun H. Parekh, Matthijs K. C. Hesselink

**Affiliations:** 1 Department of Nutrition and Movement Sciences, NUTRIM School for Nutrition and Translational Research in Metabolism, Maastricht University Medical Centre+, Maastricht, the Netherlands; 2 Department of Biology and Biological Engineering, Division of Chemical Biology, Chalmers University of Technology, Gothenburg, Sweden; 3 Department of Biomedical Engineering, University of Texas at Austin, Austin, TX USA; 4 Department of Molecular Spectroscopy, Max Planck Institute for Polymer Research, Mainz, Germany

**Keywords:** Athlete’s paradox, CARS microscopy, Intramyocellular lipid storage, Lipid composition, Lipid droplet chemical composition, Lipid droplets, Type 2 diabetes

## Abstract

**Aims/hypothesis:**

Intramyocellular lipid (IMCL) content associates with development of insulin resistance, albeit not in insulin-sensitive endurance-trained athletes (trained). Qualitative and spatial differences in muscle lipid composition may underlie this so-called athlete’s paradox. Here we studied triacylglycerol (TAG) composition of individual myocellular lipid droplets (LDs) in trained individuals and individuals with type 2 diabetes mellitus.

**Methods:**

Trained ($\dot{V}{\mathrm{O}}_{2\max }$ 71.0 ± 1.6 ml O_2_ [kg lean body mass (LBM)]^−1^ min^−1^), normoglycaemic (fasting glucose 5.1 ± 0.1 mmol/l) individuals and untrained ($\dot{V}{\mathrm{O}}_{2\max }$ 36.8 ± 1.5 ml O_2_ [kg LBM]^−1^ min^−1^) individuals with type 2 diabetes (fasting glucose 7.4 ± 0.5 mmol/l), with similar IMCL content (3.5 ± 0.7% vs 2.5 ± 0.3%, *p* = 0.241), but at opposite ends of the insulin sensitivity spectrum (glucose infusion rate 93.8 ± 6.6 vs 25.7 ± 5.3 μmol [kg LBM]^−1^ min^−1^ for trained individuals and those with type 2 diabetes, respectively) were included from our database in the present study. We applied in situ label-free broadband coherent anti-Stokes Raman scattering (CARS) microscopy to sections from skeletal muscle biopsies to measure TAG acyl chain length and saturation of myocellular LDs. This approach uniquely permits examination of individual LDs in their native environment, in a fibre-type-specific manner, taking into account LD size and subcellular location.

**Results:**

Despite a significant difference in insulin sensitivity, we observed remarkably similar acyl chain length and saturation in trained and type 2 diabetic individuals (chain length: 18.12 ± 0.61 vs 18.36 ± 0.43 number of carbons; saturation: 0.37 ± 0.05 vs 0.38 ± 0.06 number of C=C bonds). Longer acyl chains or higher saturation (lower C=C number) could be detected in subpopulations of LDs, i.e. large LDs (chain length: 18.11 ± 0.48 vs 18.63 ± 0.57 carbon number) and subsarcolemmal LDs (saturation: 0.34 ± 0.02 vs 0.36 ± 0.04 C=C number), which are more abundant in individuals with type 2 diabetes.

**Conclusions/interpretation:**

In contrast to reports of profound differences in the lipid composition of lipids extracted from skeletal muscle from trained and type 2 diabetic individuals, our in situ, LD-specific approach detected only modest differences in TAG composition in LD subpopulations, which were dependent on LD size and subcellular location. If, and to what extent, these modest differences can impact insulin sensitivity remains to be elucidated.

**Figure:**
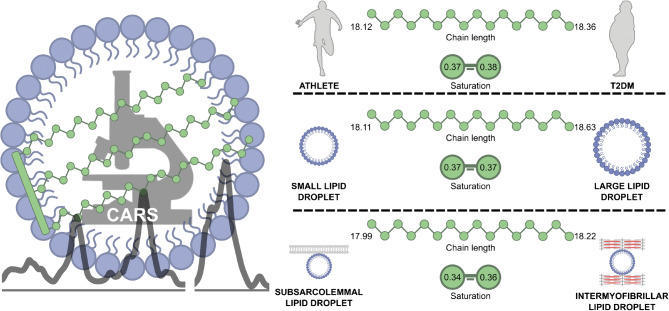
Graphical abstract

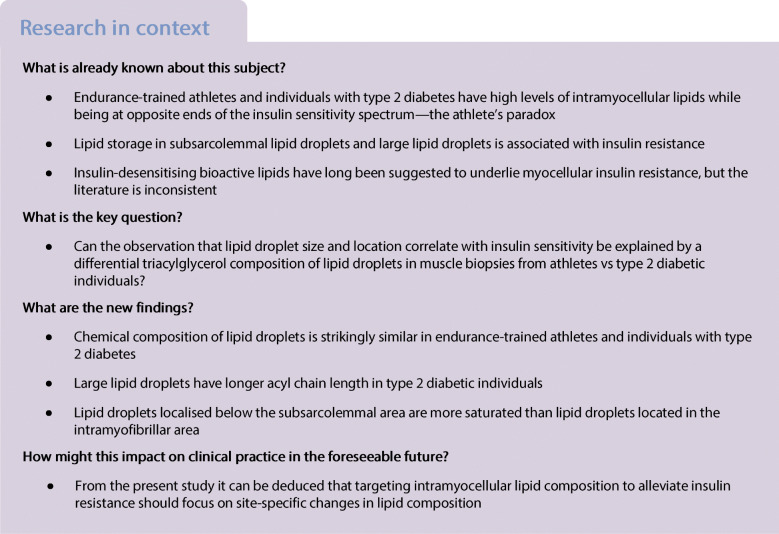



## Introduction

Excess fat can be stored ectopically as triacylglycerol (TAG) in myocellular lipid droplets (LDs) and has been associated with compromised insulin sensitivity [1–5]. Interestingly, highly insulin-sensitive trained athletes also store high amounts of myocellular fat [2, 6]. This seemingly paradoxical observation has long been suggested to originate from elevated insulin-desensitising bioactive lipids such as diacylglycerol and/or ceramides [7] in insulin-resistant muscle. However, unbiased lipidomic analysis of skeletal muscle biopsies does not consistently reveal a signature of higher levels of insulin-desensitising bioactive lipids in the muscle of individuals with type 2 diabetes compared with lean sedentary or physically trained individuals (for review see [8]).

Recently, we employed a detailed morphometric analysis of myocellular LDs in muscle biopsies obtained from individuals encompassing the complete spectrum of insulin sensitivity [6]. We observed that athletes store most of their lipids in type I muscle fibres in numerous, normally sized LDs in the intramyofibrillar (IMF) area, while individuals with type 2 diabetes store most of their lipids in fewer, but larger, LDs predominantly in the subsarcolemmal (SS) area of type II muscle fibres. Lipid storage in the SS region correlated negatively with insulin sensitivity. Upon an insulin-sensitising exercise training programme, the LD phenotype (i.e. morphology and location) in type 2 diabetic individuals shifted partly towards an athlete-like phenotype. Jointly, these data suggest that LD location and size are both correlated with insulin sensitivity.

Typically, information on LDs with respect to subcellular distribution (fibre type and SS vs IMF) and LD size is lost upon lipid extraction in conventional lipidomic analysis. Our observation that size and subcellular distribution of LDs are different between type 2 diabetic individuals and athletes stresses the need for an approach that takes size and location into account while analysing myocellular LD composition. Recently, we employed the quantitative non-linear vibrational microscopy method broadband coherent anti-Stokes Raman scattering (CARS/BCARS) microscopy to measure characteristics of lipids of single LDs [9]. In tandem with optical microscopy, label-free BCARS microscopy permits in situ quantitative analysis of the chemical characteristics specific to lipid fibre type in their native environment and with a high spatial resolution (~300 nm). Specifically, this approach permits quantitative analysis of average TAG acyl chain length and level of saturation [10]. Both acyl chain length and saturation are recognised determinants of the insulin-desensitising potency of fatty acids [11, 12].

Here we employed an in situ, label-free BCARS microscopy approach on muscle biopsy samples derived from participants spanning the physiological range of insulin sensitivity. We uniquely aimed to study whether the observation that LD size and location correlate with insulin sensitivity can be explained by a differential TAG composition of LDs in muscle biopsies from athletes vs type 2 diabetic individuals. We hypothesised that acyl chain length is longer and that the degree of saturation is higher in myocellular LDs of type 2 diabetic individuals as opposed to athletes. More specifically, we hypothesised that the acyl chain length and the degree of saturation are higher in large LDs as well as in LDs present in the SS area.

## Methods

### Participants

Eight endurance-trained athletes (trained; $\dot{V}{\mathrm{O}}_{2\max }$ >55 ml O_2_ [kg lean body mass (LBM)]^−1^ min^−1^) and eight type 2 diabetic individuals were selected from previous studies (see Vosselman et al [13] and Brouwers et al [14]). Trained and type 2 diabetic individuals were included upon matching for total intramyocellular lipid (IMCL) content as determined by the fraction of LD area over the muscle fibre area, measured previously in this cohort [6]. In short, LDs were staining with Bodipy 493/503 and imaged with widefield microscopy. The original studies were executed in agreement with the Declaration of Helsinki and approved by the Medical Ethical Committee of Maastricht University. Participants gave written informed consent before participating in the original study, registered at clinicaltrials.gov as NCT01317576.

### Muscle biopsy and metabolic measurements

Muscle biopsies and metabolic measurements have been described in more detail previously [13, 14]. After an overnight fast a biopsy was taken from the *vastus lateralis* muscle, frozen in melting isopentane and stored at −80°C. Directly after the muscle biopsy a hyperinsulinaemic–euglycaemic clamp (40 mU insulin, 5.5 mmol/l glucose) was performed and glucose infusion rate (GIR) was determined as a measure for whole body insulin sensitivity. To determine $\dot{V}{\mathrm{O}}_{2\max }$, a graded maximal cycling test with indirect calorimetry (Omnical, Maastricht, the Netherlands) was executed until exhaustion. GIR and $\dot{V}{\mathrm{O}}_{2\max }$ are expressed per kg LBM to correct for differences in fat mass. Body composition was measured with a dual-energy x-ray absorptiometry (DXA) scan.

### BCARS microscopy

LD composition was measured in a fibre-type-specific fashion. Four μm sections were stained against laminin (L9393, Sigma, St Louis, USA) and myosin heavy chain type I (A4.840, Developmental Studies Hybridoma Bank, Iowa City, IA, USA) [6]. Scout images of the sections were acquired on an Olympus IX80 inverted microscope for muscle fibre typing prior to CARS imaging [9]. In short, a dual-output laser source (Leukos-CARS, Leukos) provided a 1064 nm beam at 32 kHz repetition rate. That beam was split into the pump/probe (1064 nm) and Stokes beams (1100–1800 nm via a photonic crystal fibre and long-pass filter). Both lasers were routed on an optical table to excite the sample mounted on a stepper-motor stage (Microstage, Mad City Labs, Switzerland) with a nested piezo stage (Nano-PDQ 375 HS, Mad City Labs). Type I and type II fibre area was scanned in nine tiles of 25× 25 μm. Three fields of view per participant were imaged with 250 nm pixel size. CARS spectra were acquired between 600 and 3400 cm^−1^ and detected by a deep-depletion CCD (Newton DU920P-BR-DD, Andor, Oxford Instruments, Abingdon, United Kingdom) using custom written software (Labview, National Instruments).

### Extraction of characteristic LD spectra

CARS spectra were analysed using IgorPro 6.37 (Wavemetrics; https://www.wavemetrics.com/) and converted into Raman-like (RL) spectra [15, 16]. CH_2_-intensity based images were constructed by integrating the RL signal from 2840 to 2855 cm^−1^. In these CH_2_ images, regions of interest (ROIs) were created for further analysis of the LDs using an iterative threshold. The RL spectra in these pixels were reconstructed using five components as described by Paul et al [10]. These components were derived from RL spectra of a set of known TAG standards and their mixture (TAG backbone, TAG acyl chain length, TAG number of double bonds), a BSA RL-spectrum (generic protein), and TAGs diluted in organic solvents (TAG dilution). These data were combined to retrieve the acyl chain length and number of double bonds per acyl chain (C=C number) of unknown TAGs in the LDs.

### Calculating average acyl chain lengths and C=C number in LDs

Images of CH_2_ intensity, acyl chain length and C=C number map were analysed in ImageJ 1.51 j8 (NIH, Bethesda, USA). ROIs for fibre types (as identified by fluorescence labelling) were drawn from the CH_2_ images and applied to the acyl chain length and C=C number maps to obtain fibre-type-specific data. Acyl chain length and C=C number dependence on LD size was determined specifically for the type 2 diabetic individuals, as we previously observed that these individuals display large LDs [6]; LDs <4 pixels in area were excluded from analysis. Thus, a histogram of all LD sizes for each individual was generated with the lower 50% being defined as ‘small’ LDs (on average <1.17 μm^2^) and the upper 10% as ‘large’ (on average >3.45 μm^2^). This permitted the computation of the average acyl chain length and C=C number for small and large LDs. Similarly, acyl chain length and C=C number were assessed for LDs in the SS vs the IMF region [6]. Thus, average acyl chain length and C=C number were calculated as the weighted average of the values of type I and II fibres [6]. Group means per fibre type were computed by averaging all spectra per individual and subsequently computing the mean of this average over all athletes or type 2 diabetic individuals in the respective groups.

### Statistics

Data are presented as mean ± SD unless stated otherwise. Statistical analyses were performed using SPSS version 21.0 (SPSS, Chicago, IL, USA). Participant characteristics as well as acyl chain length and C=C number were compared between groups using an independent samples *t* test. SS and IMF LDs as well as large and small LDs were compared for acyl chain length and C=C number using a paired samples *t* test. A *p* value <0.05 was considered to be statistically significant.

## Results

### Participant characteristics

Participant characteristics have previously been reported [13, 14] and are now shown in Table 1. Type 2 diabetic individuals were significantly older and had a higher body mass, percentage fat mass and BMI than trained individuals (all *p* < 0.05). As expected, trained individuals had higher insulin sensitivity (expressed by GIR) and $\dot{V}{\mathrm{O}}_{2\max }$ and lower fasting plasma glucose levels than type 2 diabetes individuals (all *p* < 0.05). By design, trained and type 2 diabetic individuals had similar IMCL content (3.5 ± 0.7% vs 2.5 ± 0.3%, *p* = 0.241).

**Table 1:** Participant characteristics

Variable	Trained	T2DM
Age (years)	26.0 ± 1.8	60.6 ± 2.0^*^
Body weight (kg)	72.4 ± 2.6	95.9 ± 2.8^*^
% Fat mass	13.4 ± 0.5	28.3 ± 0.9^*^
BMI (kg/m^2^)	21.0 ± 0.6	29.6 ± 0.8^*^
Fasting glucose (mmol/l)	5.1 ± 0.1	7.4 ± 0.5^*^
GIR (μmol [kg LBM]^−1^ min^−1^)	93.8 ± 6.6	25.7 ± 5.3^*^
$\dot{V}{\mathrm{O}}_{2\max }$ (ml O_2_ [kg LBM]^−1^ min^−1^)	71.0 ± 1.6	36.8 ± 1.5^*^
IMCL (%)	3.49 ± 0.69	2.48 ± 0.29
Fasting insulin (pmol/l)	–	107.7 ± 25.7
HOMA-IR (%)	–	5.5 ± 1.0
Glucose-lowering medication (number of individuals)		Metformin (*n* = 7)
		DPP-4 inhibitor (*n* = 2)
		Sulfonylurea (*n* = 2)

Data are mean ± SEM, *n* = 8 for each group, except where shown otherwise

*p < 0.05 vs Trained

T2DM, type 2 diabetes mellitus

### Overall acyl chain length and saturation is not different between trained and type 2 diabetic individuals

**Fig. 1:**
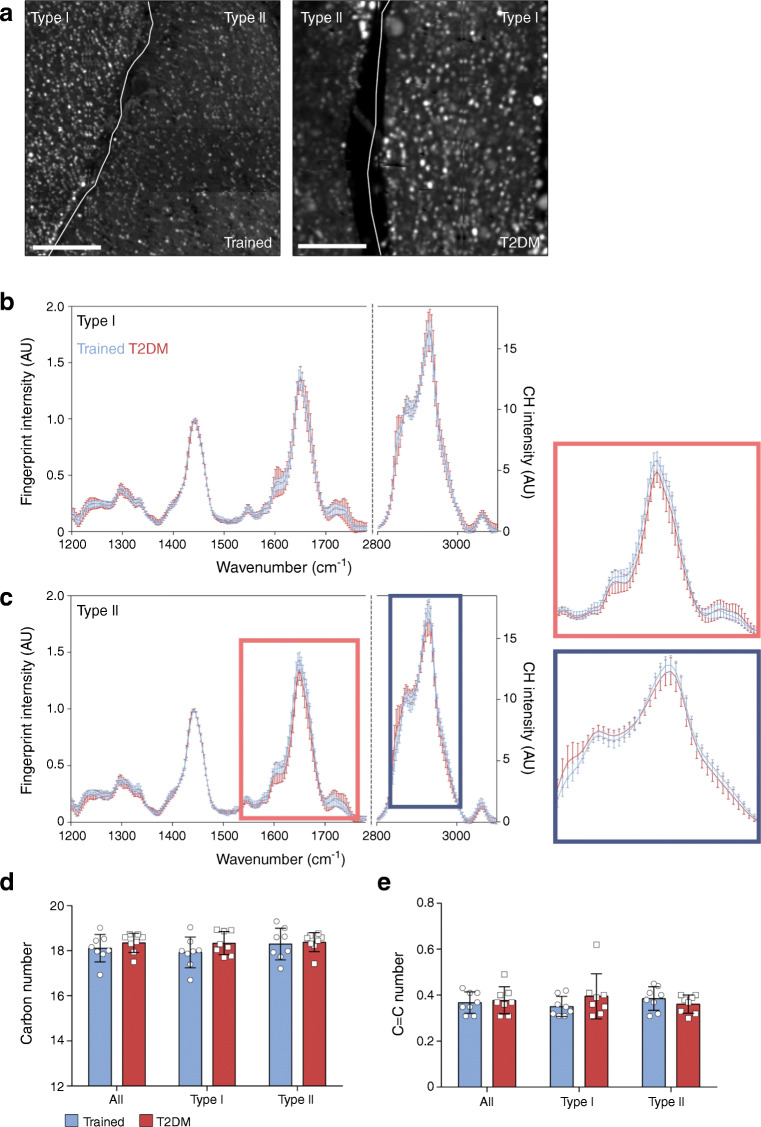
TAG chain length and saturation of LDs does not differ between trained and type 2 diabetic individuals. (**a**) Representative images of the integrated CH_2_ intensity (2840–2855 cm^−1^), revealing the LDs in type I and type II fibres of trained and type 2 diabetic (T2DM) individuals, with fibre borders indicated in white; scale bars, 20 μm. (**b**, **c**) Average CARS spectra of LDs of trained and type 2 diabetic individuals, showing fingerprint intensity and intensity in the CH region (2800–3100 cm^−1^), in type I (**b**) and type II (**c**) fibres (mean ± SD); the enlarged, framed peaks in (**c**) have been broadened to show differences and similarities more clearly. (**d**) Number of carbons per acyl chain and (**e**) number of double bonds per acyl chain (C=C number) within LDs for all fibres, type I fibres and type II fibres of trained and type 2 diabetic individuals (mean ± SD). *n =* 8 for each group

We first aimed to identify, in a fibre-type-dependent fashion, whether TAG acyl chain length and saturation of myocellular LDs differed between trained and type 2 diabetic individuals. LDs could be readily identified based on CH_2_ intensity images (Fig. 1a) owing to a high abundance of acyl chains in LDs. Over the entire spectral range, the average RL spectra of trained and type 2 diabetic individuals were strikingly similar. This similarity was observed in type I and type II fibres (Fig. 1b, c), indicating that the chemical composition of the LDs in trained and type 2 diabetic individuals was similar. More specifically, this was observed for the CH region (2800–3100 cm^−1^), as well as for the ‘fingerprint region’ (700–1750 cm^−1^), which includes characteristic resonances of complex macromolecules, including lipids and proteins. From the average spectra for each group, average acyl chain length and average number of double bonds per acyl chain (C=C number, representing the level of unsaturation) of TAGs was determined. This way of computing can result in fractional data for acyl chain length and number of double bonds; e.g. if a TAG molecule is composed of two C16:0 acyl chains and one C18:1, CARS will give C16.67:0.33 as average chain length and number of double bonds. In contrast to what was anticipated, no differences were observed between acyl chain length in trained and type 2 diabetic individuals (18.12 ± 0.61 vs 18.36 ± 0.43 number of carbons for trained and type 2 diabetic individuals, respectively; *p* = 0.38, Fig. 1d). When examined in a fibre-type-specific fashion, acyl chain length in type I fibres of trained individuals was similar to acyl chain length measured in type 2 diabetic individuals (17.93 ± 0.68 vs 18.34 ± 0.51 carbon number for trained and type 2 diabetic individuals, respectively; *p* = 0.20, Fig. 1d). A similar observation was made for acyl chain length in type II fibres (18.30 ± 0.70 vs 18.38 ± 0.42 carbon number for trained and type 2 diabetic individuals, respectively; *p* = 0.80, Fig. 1d).

As a reflection of the level of saturation of the acyl chains, the number of double bonds per acyl chain was measured. We observed that the number of double bonds was similar in trained vs type 2 diabetic individuals (0.37 ± 0.05 vs 0.38 ± 0.06 C=C number for trained and type 2 diabetic individuals, respectively; *p* = 0.71, Fig. 1e). This lack of difference in saturation between trained and type 2 diabetic individuals was observed in type I (0.35 ± 0.04 vs 0.40 ± 0.10 C=C number for trained and type 2 diabetic individuals, respectively; *p* = 0.27, Fig. 1e) as well as in type II fibres (0.39 ± 0.05 vs 0.36 ± 0.04 C=C number for trained and type 2 diabetic individuals, respectively; *p* = 0.31, Fig. 1e).

### Region specific differences in acyl chain length and saturation of myocellular LDs

**Fig. 2:**
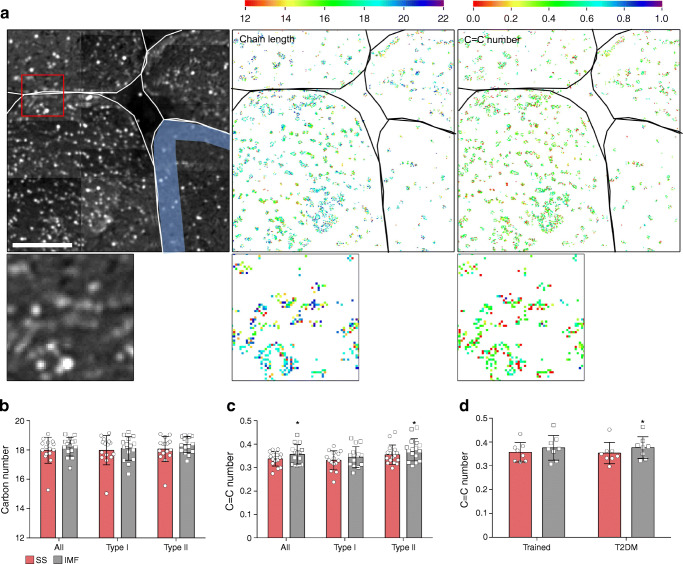
Saturation is higher in SS LDs. (**a**) CH_2_ intensity image (left) and spatial maps of chain length (middle) and C=C number (right) of a representative image of a trained participant, with magnified images below (indicated in the original image by a red box; note that the resolution of the zoomed images is the maximum possible, given the system used). Colour scales represent number of carbons per acyl chain (middle) or C=C number (right). In these images the SS region was identified by using an individual predetermined SS diameter (as illustrated by the blue area in the CH_2_ intensity image). Fibre borders are indicated in white and black; scale bars, 20 μm. (**b**, **c**) Acyl chain length (**b**) and C=C number (**c**) of SS and IMF LDs in all fibres, type I fibres and type II fibres of all participants; *n* = 16 (mean ± SD). (**d**) C=C number in LDs in type II fibres separated for trained and type 2 diabetic (T2DM) individuals; *n =* 8 for each group (mean ± SD). *p < 0.05 vs SS in the same group

SS lipid storage is considered detrimental for insulin sensitivity [6, 17–19]. In support of this, we previously observed a negative correlation between insulin sensitivity and SS LD size and number over a wide range of insulin sensitivity [6]. The current approach allows for creating spatial maps of lipid chemistry: acyl chain length and C=C number of SS and IMF LDs (Fig. 2a) and relate this to the insulin sensitivity spectrum encompassed by trained and type 2 diabetic individuals. When examining the combined muscle fibre types and pooled groups, acyl chain length in SS were non-significantly shorter than in IMF LDs (17.99 ± 0.89 vs 18.22 ± 0.64 carbon number for respectively SS and IMF, *p* = 0.077, Fig. 2b). This modest effect on acyl chain length was specific for type II fibres (18.07 ± 0.86 vs 18.37 ± 0.55 carbon number for SS and IMF, respectively; *p* = 0.06, Fig. 2b) and not for type I fibres (17.99 ± 1.00 vs 18.12 ± 0.81 carbon number for SS and IMF, respectively; *p* = 0.31, Fig. 2b). Interestingly, the number of double bonds was significantly, albeit modestly, lower in the SS region compared with the IMF region when the groups were pooled (0.34 ± 0.02 vs 0.36 ± 0.04 C=C number for SS and IMF, respectively; *p* < 0.05, Fig. 2c). This lower C=C number was observed in the mixed fibre population, but was driven by differences specifically in type II fibres (0.35 ± 0.04 vs 0.38 ± 0.05 C=C number for SS and IMF, respectively; *p* < 0.05, Fig. 2c).

Next, we examined whether the spatial differences in C=C number that were detected in type II fibres over the entire range of insulin sensitivity also was detectable if trained and type 2 diabetic individuals were examined separately. Interestingly, we observed a significantly lower C=C number in SS LDs compared with IMF LDs in type II fibres in type 2 diabetic individuals (0.35 ± 0.04 vs 0.38 ± 0.05 C=C number for SS and IMF, respectively; *p* < 0.01, Fig. 2d), but not in trained individuals (0.36 ± 0.04 vs 0.38 ± 0.05 C=C number for SS and IMF, respectively; *p* = 0.31, Fig. 2c). Thus, over the entire range of insulin sensitivity, acyl chains in the IMF region had more double bonds than in the SS region, an observation made in type II, but not in type I fibres. Upon separating the trained and the type 2 diabetic samples, the difference in double bonds between the SS and IMF LDs was only detected in the type 2 diabetic individuals.

### Higher acyl chain length in large LDs

**Fig. 3:**
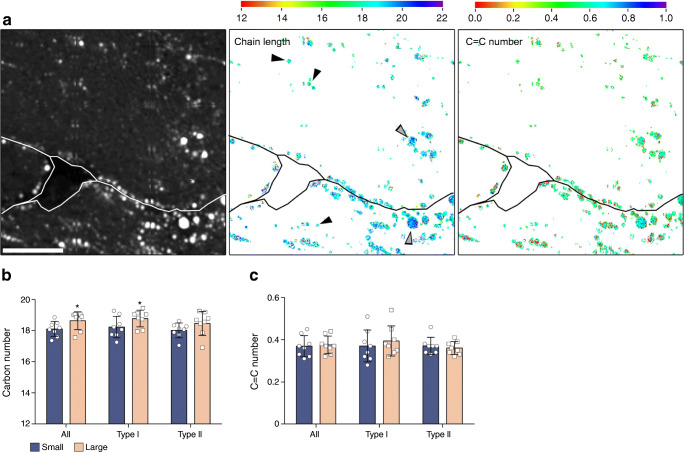
Higher chain length in large LDs. (**a**) CH_2_ intensity image (left) and spatial maps of chain length (middle) and C=C number (right) of a representative image of a type 2 diabetic (T2DM) participant. Colour scales represent number of carbons per acyl chain (middle) or C=C number (right). Small LDs indicated by black arrows, large LDs indicated by grey arrows. Fibre borders are indicated in white and black; scale bars, 20 μm. (**b**, **c**) Acyl chain length (**b**) and C=C number (**c**) of small and large LDs in all fibres, type I fibres and type II fibres (mean ± SD) *p < 0.05 vs small LDs in the same group; *n* = 8 for large and small LDs

We previously reported in the same cohort that LDs are larger in type II fibres of type 2 diabetic individuals than in trained individuals and observed a negative correlation between LD size and insulin sensitivity [6]. Thus, we specifically aimed to investigate whether large LDs in type 2 diabetic individuals differed in acyl chain length and saturation compared with the more frequently present smaller sized LDs. The spatial maps of acyl chain length and C=C number allowed us to directly relate LD size to acyl chain length and C=C number (Fig. 3a). Large LDs were defined as the 10% largest LDs and small LDs as the 50% smallest LDs present in the type 2 diabetic individuals. We performed analysis of acyl chain length and C=C number in relation to LD size only in type 2 diabetic individuals, because in the trained individuals we found hardly any LDs that are as large as the largest 10% LDs in the type 2 diabetic individuals. Interestingly, we observed that acyl chains of large LDs were significantly longer compared with small LDs, upon examining both type I and type II muscle fibres (18.11 ± 0.48 vs 18.63 ± 0.57 for small and large LDs, respectively; *p* < 0.05, Fig. 3b). This difference is visualised in the spatial maps, where large LDs often displayed longer acyl chains than small LDs (Fig. 3a, indicated by the grey arrows vs black arrows, respectively). We observed that the difference in LD composition between small and large LDs predominantly originated from LDs in type I fibres (18.23 ± 0.69 vs 18.77 ± 0.44 carbon number for small and large LDs, respectively; *p* < 0.05, Fig. 3b). For type II fibres statistical significance was not reached (18.01 ± 0.48 vs. 18.46 ± 0.77 carbon number for small and large LDs, respectively; *p* = 0.25, Fig. 3b). The number of double bonds was similar between large and small LDs (all fibres: 0.37 ± 0.05 vs 0.37 ± 0.04 C=C number for small and large, respectively; *p* = 0.70, Fig. 3c). Thus, the large LDs present in type 2 diabetic individuals have a longer acyl chain length compared with smaller, normally sized LDs in the same patient population.

## Discussion

This study is the first to compare the composition of individual myocellular LDs between two populations representing the two extremes of the insulin sensitivity spectrum in a spatial and fibre-type-specific fashion. Given the notable differences in insulin sensitivity between trained and type 2 diabetic individuals, and the putative insulin-desensitising effects of intramyocellular lipids, we were anticipating that we would detect profound differences in composition of myocellular LDs in skeletal muscle biopsies of trained vs type 2 diabetic individuals. The composition of LDs with respect to chemical bonds that are detectable with BCARS, however, was remarkably similar for trained and type 2 diabetic individuals. Specifically, length and saturation of the acyl chains within LDs were not different between trained and type 2 diabetic individuals. We previously observed, however, that it is not overall LD content, but specific subpopulations of LDs (i.e. large LDs and SS LDs), that associate with insulin resistance [6]. Thus, we took advantage of the unique ability of in situ chemical imaging with the BCARS microscopy approach to analyse the composition of individual LDs in a muscle-fibre-type specific fashion and in relation to LD size and spatial distribution. Spatial analysis of acyl chain length and C=C number revealed the novel insight that acyl chains found in LDs in the SS area were shorter, albeit not statistically significant, and more saturated than acyl chains in IMF LDs. Furthermore, acyl chains in large LDs (typically correlated with lower insulin sensitivity) in type 2 diabetic individuals typically are longer than acyl chains in smaller, normally sized LDs. The differences in composition are more distinct in type II rather than in type I muscle fibres, in SS rather than in IMF LDs, and in large rather than small LDs. All these variables have previously been reported to relate to low rather than high insulin sensitivity [6, 17, 18]. Nevertheless, it should be noted that the differences in acyl chain length and saturation appear only modest (albeit significant) and only in a subpopulation of LDs and that it remains unclear if these differences indeed contribute to the development of insulin resistance.

Previous studies have indicated that acyl chain length and saturation of TAGs in the plasma are associated with risk for diabetes [20] as well as improvements in insulin sensitivity after diet-induced weight loss [21]. Specifically in the muscle, it has been reported that saturated TAGs negatively correlate with insulin sensitivity when measured after lipid extraction from muscle [22]. Furthermore, an increased saturated acyl content of TAGs was reported in skeletal muscle of normoglycaemic obese participants compared with normoglycaemic lean participants [22]. The present study reveals that the increased saturation that was previously reported most likely does not originate from acyl chains found in LDs as we did not detect differences in acyl chain length or saturation in the overall LD pool between trained and type 2 diabetic individuals.

Impaired insulin signalling in insulin-resistant muscle has long been attributed to (elevated) presence of bioactive lipid derivatives, commonly thought to originate from excess lipid storage in muscle [23]. Bioactive lipids of a wide variety of species are commonly measured by lipidomics. Lipidomic approaches require lipid extraction of the sample followed by mass spectrometry to quantify the individual lipid species. However, upon extracting lipids from their native environment, all spatial information is lost. LD location appears to be a determinant (or at least reflective) of insulin sensitivity. Specifically, lipids in the SS region have been proposed to affect insulin sensitivity as lipids can readily interfere with insulin signalling at the cell membrane, owing to their close proximity. We and others observed a negative correlation between insulin sensitivity and SS LDs [6, 17, 18]. Thus, we aimed to investigate whether this correlation could result from a differential composition between SS and IMF LDs. We observed that SS LDs were modestly but significantly more saturated compared with acyl chains in IMF LDs in type II fibres. Although this difference was most pronounced in the type 2 diabetic individuals, we did not observe differences in the composition of SS LDs nor IMF LDs between trained and type 2 diabetic individuals. However, we previously reported that lipid storage in the SS region of type II muscle fibres is almost fourfold higher in type 2 diabetic individuals compared with trained athletes [6]. Thus, this higher SS lipid storage in the muscle of type 2 diabetic individuals can contribute to an elevated storage of saturated acyl chains in the SS region of type 2 diabetic compared with trained individuals. Lipids in the SS regions have previously been associated with compromised insulin sensitivity. It has, for example, been shown that the fatty acid composition of phospholipids in the sarcolemma may affect insulin sensitivity; specifically the degree of unsaturation correlated positively with insulin sensitivity [24]. Likewise, specifically ceramides located in the sarcolemma [25] and membrane-associated saturated diacylglycerols associated negatively with insulin sensitivity [19]. The storage of more saturated acyl chains in SS LDs in the vicinity of the sarcolemma may contribute to alterations in lipid composition of the sarcolemma and hence impede insulin signalling.

We previously observed that type 2 diabetic individuals are characterised by large LDs in type II fibres compared with trained individuals [6]. In line with this, multiple studies have reported that LD size negatively correlates with insulin resistance [6, 17, 26]. Thus, we aimed to specifically investigate the composition of these large LDs observed in type 2 diabetes individuals. We compared acyl chain length and saturation of large LDs to smaller, normally sized LDs and observed that in large LDs acyl chains are longer than in small LDs. Thus, the higher prevalence of large LDs in type 2 diabetic individuals as opposed to trained athletes indicates an increased presence of longer acyl chains. This observation is in line with previous cell and animal studies showing that fatty acids with a longer chain length contribute to insulin resistance, as opposed to fatty acids with a shorter acyl chains [12, 27, 28].

Our initial hypothesis, based upon targeted or unbiased lipidomic studies of lipids extracted from muscle cells, was that we would detect more deleterious lipid moieties in myocellular LDs in muscle biopsies from individuals with type 2 diabetes than in trained individuals. Our data do not support this hypothesis and hence are not in line with (most) data on lipid composition in lipids extracted from muscle. It is important to note that we previously have employed the same CARS-based technology to examine lipid compositional differences upon overexpression of the LD coat protein perilipin 5 (PLIN5). Overexpression of PLIN5 in rat skeletal muscle and cultured myotubes resulted in augmented storage of fat in large LDs and significant alterations in composition of the lipids in these LDs [9]. At present, BCARS and other vibrational microscopy techniques are the only label-free methods that do not require any kind of lipid extraction or pre-treatment of samples that may affect lipid composition. Hence, this approach is attractive for its ability to obtain sensitive information on composition of lipids within individual LDs in their native environment. Thus, the apparent discrepancy of our data with lipidomic analyses of lipids extracted from muscle may well originate from differences in methodology. Lipids extracted from muscle not only reflect the composition of the lipids within LDs, but also contain membrane lipids and membrane-associated lipids, which by virtue of their cellular location, are more prone to impede insulin signalling than lipids present within LDs. To examine if indeed these methodological differences underlie the apparent discrepancy between the LD-specific data in the present and other studies, future studies should combine in situ analysis with mass spectrometry, such as secondary ion mass spectrometry (SIMS) or matrix-assisted laser desorption/ionisation mass spectrometry imaging (MALDI-MSI); this will also provide more information on the lipid species involved. The recent introduction of nano-SIMS may even bring the spatial resolution down to a level that permits examination of individual LDs in situ [29].

The lack of a normoglycaemic, young, lean and untrained group for comparison with the athletes, and a normoglycaemic group that was BMI- and age-matched to the individuals with type 2 diabetes, could be considered a limitation of the present study. Instead of matching to these phenotypic differences, we matched for total IMCL content, which, irrespective of age or leanness, is considered to be the culprit for myocellular insulin resistance. Thus, the effect of IMCL on insulin sensitivity can be compared directly in two groups representing both extremes of the insulin sensitivity spectrum.

In conclusion, in situ label-free analysis of acyl chain length and saturation of individual myocellular LDs by CARS microscopy reveal a remarkable similarity in composition of LDs of insulin-sensitive trained individuals and type 2 diabetic individuals. This method of analysis allowed us to directly relate lipid composition to subcellular location and size of LDs. Higher acyl chain length or saturation could be detected in subpopulations of LDs, i.e. large LDs and SS LDs, respectively, which are more abundant in type 2 diabetic individuals and have been linked to insulin resistance. However, if and to what extent these modest differences in acyl chain length and saturation (which were only detected in LD subpopulations), can indeed impact insulin sensitivity remains to be elucidated.

## Data Availability

Data will be shared upon consultation with the corresponding author.

## Abbreviations

BCARS: Broadband coherent anti-Stokes Raman scattering
CARS: Coherent anti-Stokes Raman scattering
GIR: Glucose infusion rate
IMCL: Intramyocellular lipid
IMF: Intramyofibrillar
LBM: Lean body mass
LD: Lipid droplet
PLIN5: Perilipin 5
RL: Raman-like
ROI: Region of interest
SIMS: Secondary ion mass spectrometry
SS: Subsarcolemmal
TAG: Triacylglycerol
